# Hybrid Bead Air Filters with Low Pressure Drops at a High Flow Rate for the Removal of Particulate Matter and HCHO

**DOI:** 10.3390/polym14030422

**Published:** 2022-01-21

**Authors:** Hee Ju Kim, Ye Jin Kim, Yu Jin Seo, Ji Hee Choi, Hye Young Koo, Won San Choi

**Affiliations:** 1 Department of Chemical and Biological Engineering, Hanbat National University, 125 Dongseodaero, Yuseong-gu, Daejeon 305-719, Korea; kimhj0924@naver.com (H.J.K.); agoqkfkrl@naver.com (Y.J.K.); 6285999@naver.com (Y.J.S.); wlgml1350@naver.com (J.H.C.); 2 Functional Composite Materials Research Center, Jeonbuk Institute of Advanced Composite Materials, Korea Institute of Science and Technology (KIST), 92 Chudong-ro, Bongdong-eup, Wanju-gun, Seoul 136-791, Korea; koohy@kist.re.kr

**Keywords:** particulate matters, air filters, HCHO, removal efficiency, pressure drop

## Abstract

A tower air filtration system was designed in which bead air filters (BAFs) were actively rotated by a fan motor to remove particulate matter (PM) or HCHO gas. Three types of BAF, hydrophilic, hydrophobic, and hybrid, were prepared and compared for the removal of PM and HCHO gas. A tower air filtration system loaded with hybrid BAFs purified 3.73 L of PM (2500 μg/m^3^ PM_2.5_) at a high flow rate of 3.4 m/s with high removal efficiency (99.4% for PM_2.5_) and a low pressure drop (19 Pa) in 6 min. Against our expectations, the PM_2.5_ removal efficiency slightly increased as the air velocity increased. The hybrid BAF-200 showed excellent recyclability up to 50 cycles with high removal efficiencies (99.4–93.4% for PM_2.5_). Furthermore, hydrophilic BAF-200 could permanently remove 3.73 L of HCHO gas (4.87 ppm) and return the atmosphere to safe levels (0.41–0.31 ppm) within 60 min without any desorption of HCHO gas.

## 1. Introduction

When particulate matter (PM)-laden air passes through a fixed air filter, the PM is filtered, and a pressure drop occurs because the fixed air filter acts as a barrier to the incoming air. The pressure drop is inevitably generated by the fixed air filter during air filtration. The minimization of this pressure drop is one of the major challenges for ideal air filters because a high pressure drop causes high energy consumption. Thus, various air filters have been developed to address the pressure drop [1,2,3,4]. However, most of the abovementioned air filters are still fixed, regardless of the structure and function of the air filter. A lottery draw machine-inspired movable air filter system that deviates from typical air filters was recently reported for the removal of PM [5]. This air filter showed excellent removal efficiency and an extremely low pressure drop because of its unique air filter structure. However, the development of novel air filters that are not fixed is still attractive.

PM is a hydrophilic chemical mixture consisting of nitrate, sulfate, silicate, chloride, and elemental carbon, which are generally formed by burning fossil fuels [1]. Therefore, much effort has been devoted to developing highly polar superhydrophilic or hydrophilic air filters [6,7,8,9,10,11,12,13]. Relatively little attention has been focused on hybrid air filters composed of hydrophilic and hydrophobic materials. Only a few groups have focused on hybrid air filters with enhanced removal performance under high relative humidity and general conditions [14,15,16]. The role of hydrophobicity in hybrid air filters has not been extensively studied.

Formaldehyde (HCHO) is another target for removal by air filters. It is a highly toxic gas emitted from many industrial products. Humans who are frequently exposed to formaldehyde are diagnosed with cancer at higher rates than people who are not as exposed [17,18]. The concentration of HCHO in the air is sometimes higher when air filters are used because air filters are unable to filter HCHO but are still generally used in enclosed spaces such as homes, offices, hospitals, and laboratories [10,11]. Plasma catalysis, thermal oxidation, and catalytic oxidation have been proposed to decompose HCHO into CO_2_ and H_2_O. However, these methods inevitably produce toxic byproducts, such as NO_x_, O_3_, and OH radicals, and consume large amounts of energy [19,20,21,22]. Although the adsorption method is environmentally friendly and economical, HCHO removal by adsorption on air filters has not been extensively studied. Thus, it is of great significance to develop multifunctional air filters with high removal efficiency and a low pressure drop for the removal of PM and HCHO.

Herein, we report a tower air filtration system in which bead air filters (BAFs) are actively rotated by a fan motor to remove PM or HCHO gas. Three types of BAFs, hydrophilic, hydrophobic, and hybrid BAFs, are prepared and compared for the removal of PM and HCHO gas.

## 2. Experimental Section

### 2.1. Materials

Sulfate (melamine-formaldehyde sponge) MFS and polydimethylsiloxane (PDMS) (Sylgard 184) were purchased from BASF and Dow Chemical, respectively. Polyvinyl alcohol (PVA) (Mw: 130,000 Da) and n-hexane (C_6_H_14_, 95%) were purchased from Sigma-Aldrich. Konjac glucomannan (KGM) was purchased from Zhejiangs. All chemicals were used without further purification. Deionized (DI) water with a resistance of 18 MΩ cm was obtained from a Millipore Simplicity 185 system.

### 2.2. Preparation of Bead MFS

Two hundred pieces of cylindrical MFS (diameter: 0.5 cm and height: 0.5 cm) were added to a beaker containing DI water (200 mL). The inside of the beaker was coated with sandpaper, and the contents were stirred for 8 h. The bead sponge was prepared by immersion of the cylindrical MFS into a beaker containing water, followed by stirring and collision of the MFS. The MFS collided with the inside wall of the beaker, which was wrapped in sandpaper. The cylindrical MFS was gradually transformed into a bead sponge by physically etching the MFS with the sandpaper. The resulting products were washed three times with DI water and dried in an oven at 50 °C for 4 h.

### 2.3. Preparation of Hydrophilic BAFs

PVA powder (1 g) was dissolved in DI water (100 mL) under vigorous stirring at 200 °C for 2 h. Then, KGM powder (0.1 g) was added and dissolved in the resulting solution at 100 °C. Next, 100–200 MFS beads were immersed in the abovementioned PVA-KGM solution and heated in an oven at 100 °C for 3 h. The final products were washed three times with DI water and dried in an oven at 50 °C for 4 h.

### 2.4. Preparation of Hydrophobic BAFs

A PDMS solution (mass ratio) containing a cross-linker (1), prepolymer (10), and n-hexane (100) was prepared. Next, 100–200 MFS beads were immersed in the PDMS solution and heated in an oven at 100 °C for 1 h. The final products were washed three times with ethanol and dried in an oven at 50 °C for 4 h.

### 2.5. Preparation of Hybrid BAFs

Hybrid BAFs were prepared by a half-and-half mixture of hydrophilic and hydrophobic BAFs (hybrid BAF-n (n/2 MFS/PVA-KGM + n/2 MFS/PDMS).

### 2.6. PM and VOC Filtration

PM was generated by burning incense. The PM removal test chamber (3.63 L) was filled with low (500 μg/m^3^), medium (1000 μg/m^3^), and high (2500 μg/m^3^) concentrations of PM_2.5_. The air velocities generated by the fan motor were 2.4, 3.4, and 4.4 m/s. A constant air flow was injected into the air filter to rotate the BAFs. A handheld air quality detector was used to measure the PM concentrations before and after filtration. The removal efficiency was calculated by comparing the measured PM concentrations before and after filtration. All PM filtration tests were performed at relative humidity from 29% to 36% (standard deviation: 1.56). When air was injected into the chamber, the pressure drop was zero point-adjusted. After the addition of BAFs into the chamber and injection of air, the pressure drop was measured at the input and output sections by a differential pressure gauge. For removal of HCHO gas, the main chamber containing a tower air filtration system (middle) was connected to a glass vial (left) containing HCHO gas and a detector (right). An air velocity of 1.0 m/s was injected into the glass vial (50 mL) containing 10 μL of HCHO to push the HCHO gas from the left to the main chamber. The variation in the HCHO gas concentration emitted from tower air filtration system BAFs was monitored by an HCHO detector.

### 2.7. Characterization

SEM/EDX analyses were performed using a Hitachi S-4800 instrument. FT-IR spectra were obtained using a Sinco Nicolet IS5 instrument. TGA was performed using a thermogravimetric analyzer (Sinco TGA N-1500, Seoul, Korea) over a temperature range of 25–800 °C at a heating rate of 10 °C min^−1^ in air (flow rate, 60 cm^2^ min^−1^). The contact angle measurements were carried out using a contact angle meter (SEO Phoenix 300 Touch, Yongin-si, Korea) at ambient temperature, and the volume of the probing liquid was 20 μL. After 5 min of vigorous rotation of each BAF within the glass chamber, the surface potential of each sample was measured by an electrostatic voltmeter (HSK-5008 L, Sejong, Korea). A handheld air quality detector (HT-9601, Dongguan Xintai Instrument, Dongguan City, China) was used to measure the PM concentrations before and after filtration. By comparing the PM concentrations before and after filtration, the removal efficiency (RE) can be calculated according to the following equation:

RE (%) = (C_0_ − C_1_)/C_0_ × 100%

where C_0_ (µg m^−3^) and C_1_ (µg m^−3^) refer to the PM concentrations before and after filtration, respectively. The pressure drop was measured by a differential pressure gauge (TESTO 510i, TESTO, Germany). The flow rate was measured by a flowmeter (TESTO 450i, TESTO, Titisee-Neustadt, Germany). The error ranges of removal efficiency, pressure drop, and removal time were below 1.5%, 3%, and 4%, respectively. HCHO was measured by an HCHO measuring meter (BQ16, Trotec GmbH & Co. KG, Heinsberg, Germany).

## 3. Results and Discussion

**Figure 1:**
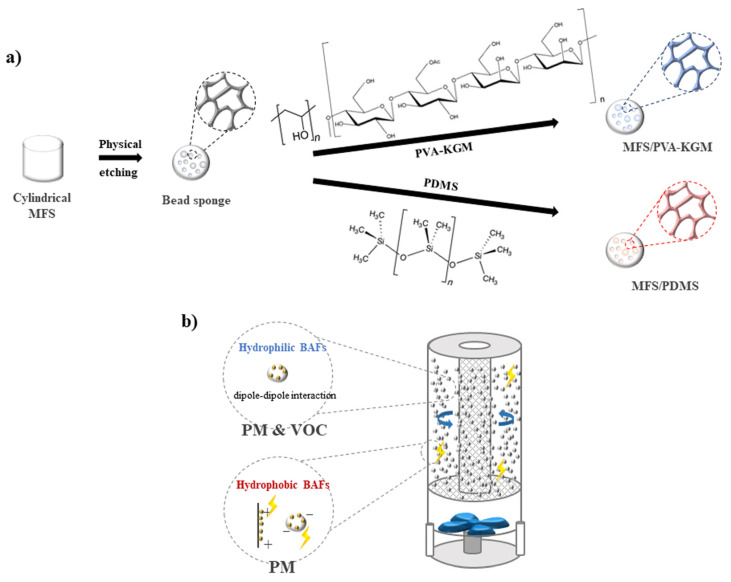
A schematic illustration of (**a**) the synthesis of two types of BAF, hydrophilic and hydrophobic, and (**b**) a tower air filtration system in which BAFs are actively rotated to capture PM or HCHO.

Figure 1a shows a schematic illustration of the synthesis of two types of BAF, hydrophilic and hydrophobic, through a physical etching of an MFS, followed by one-step coating with hydrophilic and hydrophobic polymers, respectively. Hydrophilic and hydrophobic BAFs were prepared by one-step coating of the bead sponge with a polymer solution of PVA/ KGM and PDMS, respectively. Hydrophilic, hydrophobic, or hybrid BAFs were loaded into the tower air filtration system (Figure 1b). A tower air filtration system in which hybrid BAFs are actively rotated to capture PM or HCHO is proposed.

**Figure 2:**
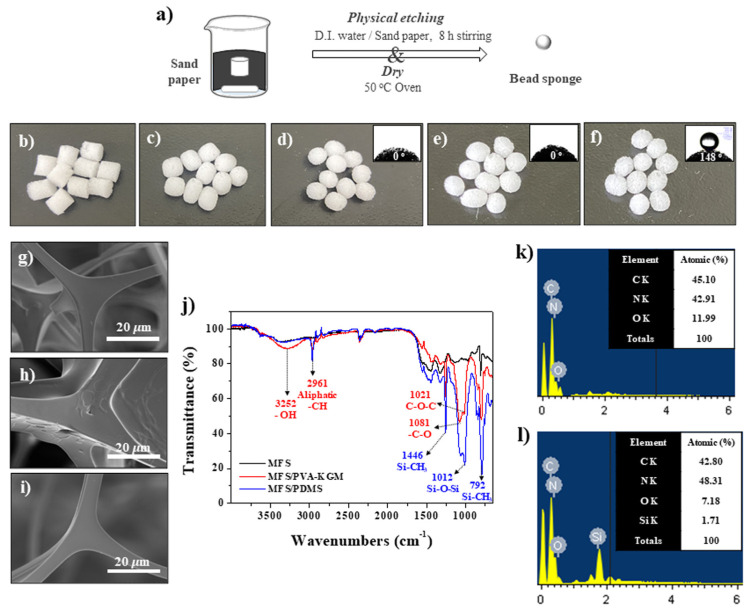
(**a**–**d**) Images showing the process of BAF formation with stirring time for (**b**) 0 h, (**c**) 4 h, and 8 h. Images of (**e**) hydrophilic BAFs (MFS/PVA-KGM) and (**f**) hydrophobic BAFs (MFS/PDMS). The insets show the corresponding WCA data. SEM images of (**g**) MFS, (**h**) hydrophilic BAFs, and (**i**) hydrophobic BAFs. (**j**) FT-IR and EDX data of (**k**) hydrophilic and (**l**) hydrophobic BAFs.

Figure 2a–d shows images of the shape variation of the cylindrical MFS while it was stirred in the beaker and wrapped in sand paper. As the stirring time increased, the sharp edge of the cylindrical MFS became round (Figure 2b–d). After 8 h, the cylindrical MFS became almost perfectly spherical (Figure 2d). The size of the resulting MFS bead decreased to 80% of the original size of the cylindrical MFS (Figure 2b,d). The sizes of the MFS beads to be used as BAFs were controlled by varying the sizes of cylindrical MFSs. MFS beads with sizes of 4 mm and 9 mm were prepared and used in this study (Figure S1). Our physical etching method enabled the mass production of homogeneous MFS beads. After the formation of the MFS beads, PVA-KGM and PDMS were separately coated onto the MFS beads to synthesize hydrophilic and hydrophobic BAFs, respectively (Figure 2e,f).

Figure 2g–i shows scanning electron microscopy (SEM) images of a sponge (MFS) bead, the hydrophilic BAF (MFS/PVA-KGM), and the hydrophobic BAF (MFS/PDMS). The sponge bead possessed an interconnected network skeleton with a smooth surface morphology (Figure 2g). The hydrophilic BAF (MFS/PVA-KGM) and hydrophobic BAF (MFS/PDMS) featured surface morphologies that were slightly more rough than that of the sponge bead, indicating that each polymer was coated on the sponge bead (Figure 2h,i). No detached fragments were observed upon handling each BAF. No color changes of hydrophilic and hydrophobic BAFs were observed after the coating of each polymer on the sponge bead (Figure 2d–f). The water contact angles (WCAs) of the sponge bead, hydrophilic BAF, and hydrophobic BAF were 0°, 0°, and 148°, indicating that MFS, MFS/PVA-KGM, and MFS/PDMS had superhydrophilic, superhydrophilic, and hydrophobic characteristics, respectively (insets of Figure 2d–f). The absorption peaks at 3252 cm^−1^ (OH), 2961 cm^−1^ (aliphatic CH), 1081 cm^−1^ (C-O), and 1021 cm^−1^ (C-O-C), the characteristic groups in the PVA-KGM spectrum [23,24,25], were increased or newly formed due to the coating of PVA-KGM for hydrophilic BAF (MFS/PVA-KGM) (red line, Figure 2j). New absorption peaks at 1446 cm^−1^ (Si-CH_3_) and 1012 cm^−1^ (Si-O-Si) were observed for hydrophobic BAFs (MFS/PDMS) (blue line) [23,24,25]. The intensity of the peak at 792 cm^−1^ related to Si-CH_3_ remarkably increased for hydrophobic BAFs (MFS/PDMS) (blue line) [23,24,25]. Energy dispersive X-ray (EDX) data also confirmed the synthesis of hydrophilic and hydrophobic BAFs (Figure 2k,l and Figure S2). The C content in the hydrophilic BAF was much higher than that in the hydrophobic BAF due to the PVA-KGM coating (Figure 2k). Si was observed in the hydrophobic BAF due to the PDMS coating (Figure 2l).

**Figure 3:**
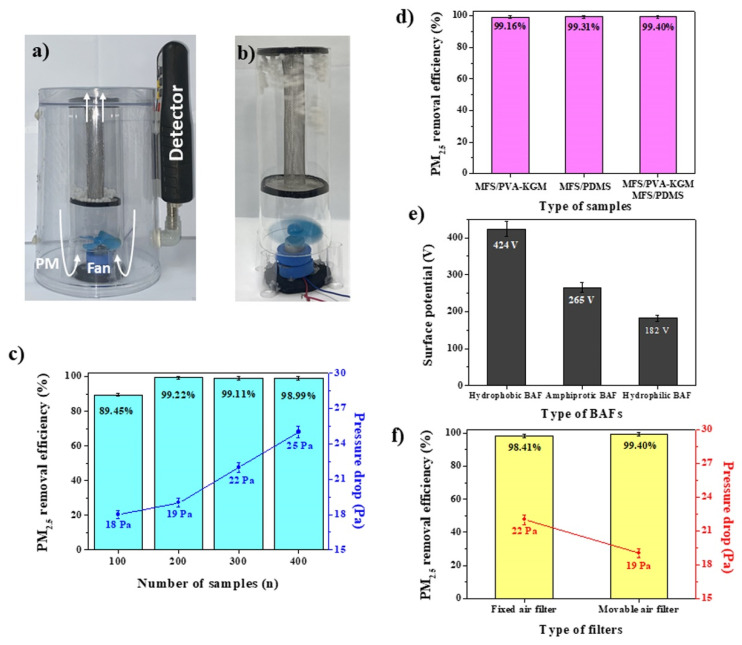
(**a**) An image of a tower air filtration system placed in an acrylate chamber. (**b**) An image of a tower air filtration system in which BAFs are actively rotated by a fan motor. (**c**) PM_2.5_ removal efficiencies and pressure drops of the air filtration systems loaded with hydrophilic BAF-100 to 400. (**d**) PM_2.5_ removal efficiencies of hydrophilic, hydrophobic, and hybrid BAF-200. (**e**) Surface potentials of hydrophilic, hydrophobic, and hybrid BAF-200. (**f**) PM_2.5_ removal efficiencies and pressure drops of the fixed BAFs (hybrid BAF-200) and movable BAFs (hybrid BAF-200).

A tower air filtration system was composed of two parts, including an air filter section containing BAFs (top) and a fan motor section (bottom) (Figure 3a). The air filter section consisted of an inner cylindrical frame (Cu mesh) and an outer chamber (glass). The BAFs can fly between the inner frame and outer chamber. A constant air flow was generated by the fan motor and injected into the air filter section to rotate the BAFs. A BAF with a diameter of 4 mm was used for the removal of PM. The tower air filtration system was placed in an acrylate chamber (3.63 L) containing a hazardous level of PM with PM_2.5_ concentrations higher than 2500 μg/m^3^. By fan motor, the PM-laden air was sucked into the bottom part of the air filtration system, passed through the filter part containing BAFs, purified, and emitted from the upper part of the air filtration system. The pressure drop was measured at input and output points by a differential pressure gauge (Figure S3). The unfiltered PM was detected in the right part of the chamber. The air velocities generated by the fan motor were 2.4–4.4 m/s, which were an order of magnitude higher than the previously reported values [12,26,27,28]. When a constant air flow was injected into the part of the air filter containing the BAFs, the BAFs vigorously moved everywhere within the inside of the air filter part, and this phenomenon was observed in all cases (Figure 3b).

Figure 3c shows the PM_2.5_ removal efficiencies of the air filtration systems loaded with different numbers of hydrophilic BAF (MFS/PVA-KGM) (100, 200, 300, and 400). The air filtration system using 100, 200, 300, or 400 BAFs was denoted BAF-100, 200, 300, or 400. The PM_2.5_ removal efficiencies of BAF-100 and BAF-200 increased, but those of BAF-300 and BAF-400 remained as analogous as that of BAF-200, which indicates that the mobilities of BAF-300 and BAF-400 were lower due to the increased bulkiness and volume (Figure 3c). In fact, the BAF-300 and BAF-400 were not uniformly dispersed within the chamber by the fan (Figure S4). The pressure drops of the BAFs increased as the number of BAFs increased due to the increased bulkiness and volume (Figure 3c). However, the pressure drop of the BAF-200, which featured the highest PM_2.5_ removal efficiency, was only 19 Pa at an air velocity of 3.4 m/s. Previously reported air filters feature much higher pressure drops than BAF-200 with values of 45–499 Pa, even at a much lower air velocity of 0.2 m/s. Because BAF-200 accounts for only 7.7% of the total volume of the air filter chamber, the injected air flow is seldom impeded by BAFs, causing the pressure drop to decrease. The time to reach the PM_2.5_ concentration below 50 μg/m^3^ was also measured for BAF-100, 200, 300, and 400. The removal time was 7 min for BAF-200 and 300 and 8 min for BAF-400 (Figure S5a). BAF-100 never reached a PM_2.5_ concentration below 50 μg/m^3^ even after 10 min. Considering the PM_2.5_ removal efficiency (99.2%), pressure drop (19 Pa), and removal time (7 min), BAF-200 was determined to be an optimized condition for hydrophilic BAFs.

To investigate the effect of the wettability of BAFs on the PM_2.5_ removal efficiency, the PM_2.5_ removal efficiency of hydrophobic and hybrid BAFs was measured and compared with that of hydrophilic BAFs. Hydrophobic BAF-200 (200 MFS/PDMS) and hybrid BAF-200 (100 MFS/PVA-KGM + 100 MFS/PDMS) were used for exact comparison. Although the samples featured analogous PM_2.5_ removal efficiencies, the removal time of hybrid BAF-200 was remarkable (Figure 3d and Figure S5b). The removal times were 6 min and 8 min for hybrid BAF-200 and hydrophobic BAF-200, respectively, which were faster and slower than that of hydrophilic BAF-200 (7 min) (Figure S5b). Based on the removal time (6 min), hybrid BAF-200 was the optimal BAF condition.

To reveal the reason why hybrid BAF-200 showed the best performance, the surface potentials of hydrophilic, hydrophobic, and hybrid BAF-200 were measured after the rotation of each BAF within the chamber for 5 min. Hydrophobic BAF-200s exhibited the highest surface potential, of 424 V, which was much higher than the values of 265 V and 182 V of hybrid BAF-200 and hydrophilic BAF-200, respectively (Figure 3e). The strongest electrostatic effect of hydrophobic BAF-200 was also observed with the naked eye after the rotation of each BAF within the chamber (hydrophobic BAF-200 > hybrid BAF-200 > hydrophilic BAF-200) (Figure S6). Through the vigorous rotation of hydrophobic BAF-200 within the glass chamber, a strong electric field can be generated through contact electrification. During the rotation of hydrophobic BAFs, the PDMS on hydrophobic BAFs generates negative charges, and the glass chamber forms positive charges according to the triboelectric series, leading to the formation of an electric field between the hydrophobic BAFs and the glass chamber. Since the PDMS of the hydrophobic BAFs and the glass of the chamber are at the opposite ends of the triboelectric series [29], hydrophobic BAFs can obtain electrons more easily than the others. The PVA of the hydrophilic BAFs is relatively close to the glass of the chamber in the triboelectric series [29], forming a weaker electric field than the PDMS of the hydrophobic BAFs. Thus, hydrophobic BAFs can efficiently eliminate PM using their strong electric fields. However, electric field by contact electrification may not be the main reason for hydrophilic BAFs because hydrophilic BAFs (MFS/PVA-KGM) possess a much lower surface potential (182 V) than hydrophobic BAFs (424 V).

The hydrophilic BAF (MFS/PVA-KGM) was composed of PVA and KGM, which feature high dipole moments (1.2 D and 3.1 D) [15,30]. These constituents can play an important role in removing PM because PM mainly consists of various hydrophilic components, such as sulfate, nitrate, silicate, calcium, chloride, and iron, and the polar groups of the constituents undergo strong dipole–dipole and induced–dipole interactions that can be used to capture PM [7,12]. Hybrid BAF-200 possessed both a high-dipole moment due to hydrophilic BAF-100 and a strong electric field due to hydrophobic BAF-100. Thus, PM can be removed by both effects. Hybrid BAF-200 showed analogous PM2.5 removal efficiency to the others (hybrid BAF-200 (99.4%) = hydrophobic BAF-200 (99.3%) = hydrophilic BAF-200 (99.1%)). Hybrid BAF-200 showed slightly higher PM_2.5_ removal efficiency than the others (hybrid BAF-200 (99.4%) > hydrophobic BAF-200 (99.3%) > hydrophilic BAF-200 (99.1%)), while it exhibited a much higher removal time performance than the others (hybrid BAF-200 (6 min) > hydrophilic BAF-200 (7 min) > hydrophobic BAF-200 (8 min)) (Figure 3d and Figure S5b). The PM_2.5_ removal efficiency (99.3%) of hydrophobic BAF-200 was analogous to that of hydrophilic BAF-200 (99.1%), while the removal time (7 min) of hydrophilic BAF-200 was shorter than that of hydrophobic BAF-200 (8 min) (Figure 3d and Figure S5b). The hydrophilic BAFs were dominant in the removal time. These results suggested that using a high dipole moment to remove PM was more suitable for lowering the removal time. Thus, the hybrid BAF, which exhibited the properties of both BAFs, offers both a fast removal time and a high PM_2.5_ removal efficiency.

To further reveal the characteristics of the hybrid BAFs, comparative experiments involving a movable hybrid BAF-200 and a fixed hybrid BAF-200 were conducted to test the removal of PM because the hybrid BAFs described here were movable. To test the fixed hybrid BAF, the hybrid BAF-200 was fixed at the bottom section of the air filter chamber (Figure S7). No movement of the hybrid BAFs was observed when PM-laden air passed through the air filter chamber. The PM_2.5_ removal efficiency was lower and the pressure drop of the fixed BAFs was higher than those of the movable BAFs, which indicated that the movable BAFs were advantageous over the fixed BAFs for the removal of PM (Figure 3f). The movable BAFs also removed the PM more quickly than the fixed BAFs (Figure S5c). The removal time to reach the PM_2.5_ concentration below 50 μg/m^3^ was 6 min for the movable BAFs, while it was 9 min for the fixed BAFs, suggesting that air filtration performances can depend on the filter type (movable or fixed BAF) even if hybrid BAFs are used in tandem. The fixed hydrophilic and hydrophobic BAFs further confirmed these results. The fixed hydrophilic and hydrophobic BAF-200 were also applied to test the removal of PM. The PM_2.5_ removal efficiencies of both fixed BAF-200 were lower than those of the movable BAF-200 (Figure 3d and Figure S8). The fixed BAFs also removed the PM more slowly than the movable BAFs (Figures S5 and S8). Although hydrophobic BAFs showed the highest specific surface area among the samples (hydrophobic BAFs (4.28 m^2^/g) > hybrid BAFs (3.93 m^2^/g) > hydrophilic BAFs (3.58 m^2^/g)), the fixed hydrophobic BAFs showed the lowest PM_2.5_ removal efficiency (Figures S8 and S9). The Brunauer–Emmett–Teller (BET) data reconfirmed that air filtration performances can depend on the filter type (movable or fixed BAF) rather than specific surface areas. The abovementioned results demonstrated the superiority of the movable BAF over the fixed BAF.

**Figure 4:**
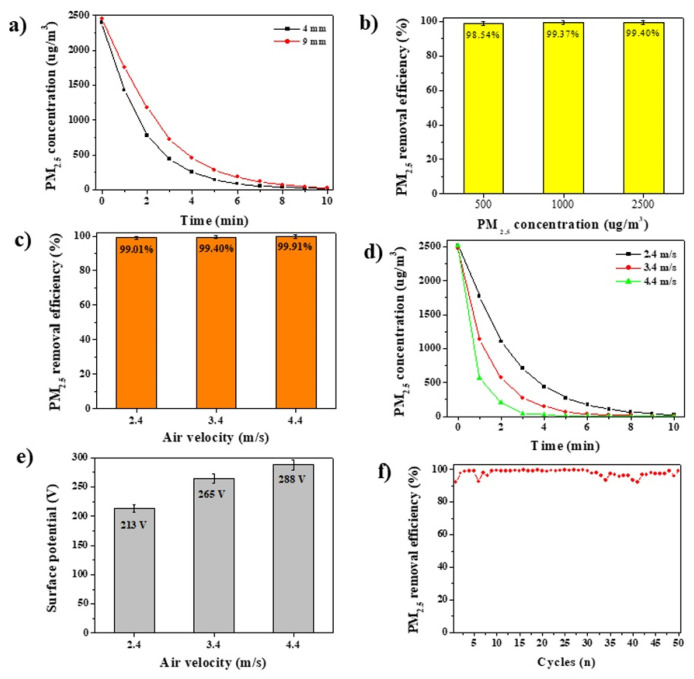
(**a**) PM_2.5_ removal efficiencies of 4 mm and 9 mm BAFs (hybrid BAF-200). (**b**,**c**) PM_2.5_ removal efficiencies of hybrid BAF-200 at different (**b**) PM_2.5_ concentrations (500, 1000, and 2500 μg/m^3^) and (**c**) air velocities (2.4, 3.4, and 4.4 m/s). (**d**) PM_2.5_ concentration variations of hybrid BAF-200 at different air velocities (2.4, 3.4, and 4.4 m/s). (**e**) Surface potentials of hybrid BAF-200 as a function of increasing air velocity (2.4, 3.4, and 4.4 m/s). (**f**) Long-term performance of hybrid BAF-200 up to the 50th cycle.

To investigate the size effect of the BAF on the PM_2.5_ removal efficiency, an hybrid BAF with a diameter of 9 mm was prepared and compared with the hybrid BAF with a diameter of 4 mm. Fewer 9 mm-sized BAFs than 4 mm-sized BAFs were used so that the total volume of the 9 mm BAFs was equivalent to that of the 4 mm BAFs. Although the total volume of the 9 mm BAFs was the same as that of the 4 mm BAFs, the 9 mm BAFs exhibited a lower PM_2.5_ removal efficiency and slower removal time than the 4 mm BAFs, which suggests that a small BAF is more favorable than a large BAF for the removal of PM due to its high mobility (Figure 4a). An hybrid BAF with a diameter below 4 mm was not tested because the 4 mm-sized BAF was the minimum size that could be synthesized. To investigate the effect of the PM concentration on the PM_2.5_ removal efficiency, the removal performance of hybrid BAF-200 was tested at low PM concentrations (500 and 1000 μg/m^3^). As the PM concentrations decreased, the PM_2.5_ removal efficiencies decreased (Figure 4b). However, hybrid BAF-200 still showed a high PM_2.5_ removal efficiency of 98.5%, even at a low concentration of 500 μg/m^3^ (Figure 4b). Extremely high PM_2.5_ concentrations of several tens of thousands to hundreds of thousands of μg/m^3^ were used for air filtration tests in previous reports, although these conditions do not meet the requirements for application in real situations. Thus, air filters must be designed and developed to capture PM at very low concentrations (several tens to hundreds of μg/m^3^).

Since the air velocity was another important parameter affecting the PM removal efficiency, the PM_2.5_ removal efficiencies were measured as the air velocity increased. The PM removal efficiency generally decreased as the air velocity increased because of the decreased contact time between the PM and the air filter. However, the PM_2.5_ removal efficiencies of hybrid BAF-200 slightly increased to 99.9% at an air velocity of 4.4 m/s, which was slightly higher than 99.0% or 99.4% at air velocities of 2.4 m/s and 3.4 m/s, respectively (Figure 4c). Against our expectations, the PM removal efficiency increased as the air velocity increased. The removal time to reach the PM_2.5_ concentration below 50 μg/m^3^ was 6 min at an air velocity of 3.4 m/s, while it was 3 min at an air velocity of 4.4 m/s (Figure 4d). The abovementioned data suggest that the PM_2.5_ removal efficiency could be enhanced even at high air velocities. To further explore this phenomenon, the surface potentials of the hybrid BAF-200 were measured under different air velocities (2.4, 3.4, and 4.4 m/s). The surface potential of hybrid BAF-200 was 213 V at an air velocity of 2.4 m/s, and it increased to 288 V at an air velocity of 4.4 m/s (Figure 4e). The surface potential gradually increased as the air velocity increased. At high air velocities, the chance of contact between PM and the BAFs is likely lower, while the chance of contact between the glass chamber and the BAFs is likely higher, leading to the formation of a stronger electric field and the removal of PM. It was speculated that the latter case was more common than the former case. The long-term performance of the hybrid BAF-200 was also tested for the practical application of the BAF. Each cycle was 10 min, including the removal (8 min) and stabilization (2 min) times. The operation time for the long-term performance test was 9 h with up to 50 cycles. Although the PM_2.5_ removal efficiencies of hybrid BAF-200 decreased to 95.9–93.4% after several cycles, the PM_2.5_ removal efficiencies remained as high as 99.4–93.4% until the 50th cycle (Figure 4f). In the absence of PMs, the weight variation of hybrid BAF-200 was monitored up to 50 cycles. After 50th cycle, the weight of hybrid BAF-200 slightly decreased to 98.8% (Figure S10).

**Figure 5:**
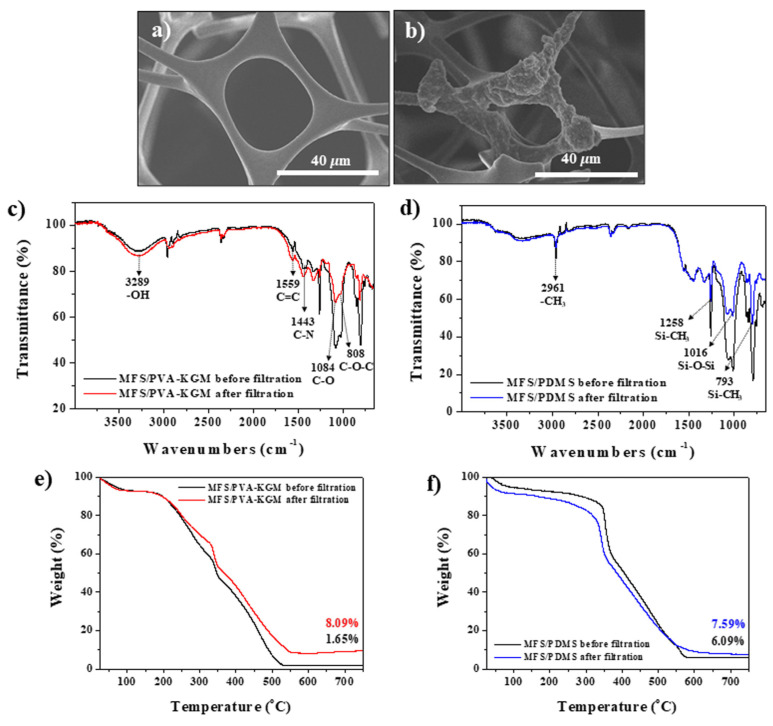
SEM images of BAFs (**a**) before and (**b**) after adsorption of PM. (**c**,**d**) FT-IR and (**e**,**f**) TGA data of (**c**,**e**) hydrophilic and (**d**,**f**) hydrophobic BAFs before and after PM filtration.

The adsorption of PM onto the hybrid BAFs was confirmed by several measurements. Figure 5a,b shows SEM images of the BAFs before and after adsorption of PM. After filtration, aggregated particles were observed on the BAF surface (Figure 5b). The BAF surface became more complicated and rough after filtration. Fourier transform infrared (FT-IR) spectroscopy was performed to further confirm the adsorption of PM. After filtration, the peak intensities at 3289 cm^−1^ (-OH stretching), 1559 cm^−1^ (C=C stretching), and 1443 cm^−1^ (C-N stretching) increased in the spectrum of the hydrophilic BAF (MFS/PVA-KGM), which indicated the adsorption of PM onto the hydrophilic BAFs because polar functional groups and elemental carbons were present at the outer surface of the PM (Figure 5c, red line) [12]. However, the peak intensities at 1084 cm^−1^ (C-O stretching) and 808 cm^−1^ (C-O-C stretching) in the spectrum of the hydrophilic BAF were lower, which suggested the adsorption of PM onto the C-O or C-O-C group of the hydrophilic BAF (Figure 5c, red line) [12]. The characteristic peaks of PDMS were lower in the spectrum of the hydrophobic BAF (MFS/PDMS) at 2961 cm^−1^ (-CH stretching), 1258 cm^−1^ (Si-CH_3_ stretching), 1016 cm^−1^ (Si-O-Si stretching), and 793 cm^−1^ (Si-CH_3_ stretching), confirming the adsorption of PM onto the hydrophobic BAFs (Figure 5d, blue line) [23,24,25]. The amount of PM adsorbed onto the hydrophilic and hydrophobic regions of the hybrid BAFs was measured by thermogravimetric analysis (TGA). The hydrophilic BAFs possessed 6.44% more mass after filtration than before filtration, while the hydrophobic BAFs possessed 1.5% more mass after filtration than before filtration, further confirming the adsorption of PM onto both hydrophilic and hydrophobic BAFs (Figure 5e,f). These TGA data also confirmed the greater adsorption of PM onto the hydrophilic BAFs than onto the hydrophobic BAFs. However, the PM_2.5_ removal efficiencies of hydrophilic and hydrophobic BAFs were 99.1% and 99.3%, respectively, which were similar to each other. These results suggested that stronger dipole–dipole and induced–dipole interactions were the driving forces of PM capture by the hydrophilic BAFs because the detected PM was mainly adsorbed on the hydrophilic BAFs. However, a strong electric field was the main cause of PM removal from the hydrophobic BAFs because the adsorption of PM was mainly observed at the inside surface of the glass chamber rather than hydrophobic BAFs. These results were in agreement with the previous surface potential data.

**Figure 6:**
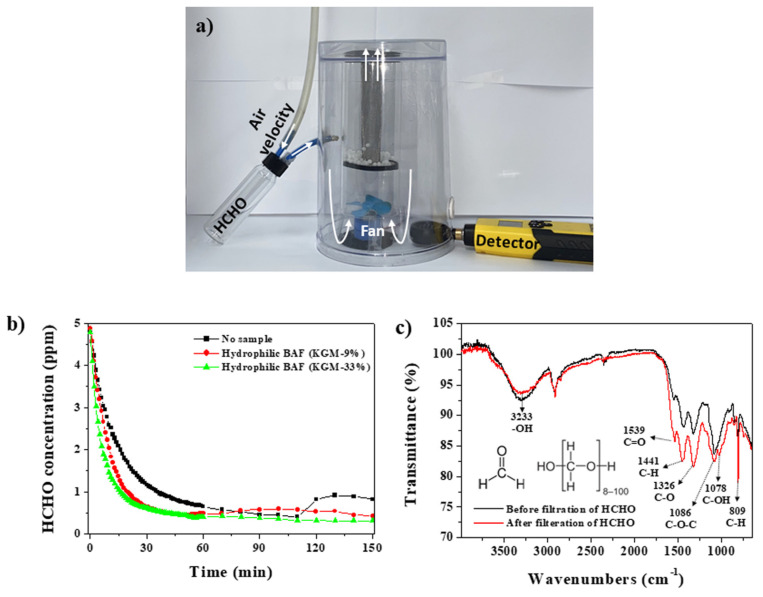
(**a**) An image showing a tower air filtration system loaded with hydrophilic BAF-200 for the removal of HCHO. (**b**) HCHO concentration variations of hydrophilic BAF-200 (KGM-9 and 33%) for 150 min. (**c**) FT-IR data of hydrophilic BAF-200 (KGM-33%) before and after HCHO filtration.

To investigate the removal ability of HCHO by hydrophilic BAF-200, a tower air filtration system loaded with hydrophilic BAF-200 was placed in a chamber (3.63 L) containing a HCHO concentration of 4.87 ppm (Figure 6a). Since the hydroxyl groups in the adsorbent can enhance the adsorption affinity of HCHO [31], hydrophilic BAFs (MFS/PVA-KGM) were tested. To further increase the adsorption affinity, two types of hydrophilic BAF (MFS/PVA-KGM, KGM-9% and 33%) were used and compared. Hydrophilic BAFs containing KGM contents over 33% were not tested because they showed poor durability as the KGM content increased. KGM contains 11 hydroxyl groups per repeating unit (Figure 1a). The variation in the HCHO gas concentration emitted from the chamber with and without the air filter was monitored as time increased. The fan was operated even in the absence of an air filter for exact comparison. In the absence of the air filter, the concentration of HCHO gas was 0.7 ppm at 60 min (black line), while it was 0.48 and 0.41 ppm at 60 min in the presence of hydrophilic BAF (KGM-9%) and BAF (KGM-33%), respectively (red and green lines, Figure 6b). Against our expectations, at 130 min, the concentrations of HCHO gas remarkably increased to 0.92 ppm when no filter was used and slightly increased to 0.54 ppm with hydrophilic BAF (KGM-9%) (black and red lines, respectively). However, the concentration of HCHO gas further decreased to 0.31 ppm with the hydrophilic BAF (KGM-33%) (green line). Thus, the net removal efficiency of the hydrophilic BAF (KGM-33%) was 66% at 130 min (Figure S11). The net removal capacities of the hydrophilic BAF (KGM-33%) and BAF (KGM-9%) for HCHO were 9.54 and 5.94 mg g^−1^, respectively; these were calculated based on the concentration ratios of the HCHO at 130 min (Figure S11). When no filter was used, HCHO could desorb from the surface of the acrylate chamber because HCHO physically adsorbed onto the inside surface of the acrylate chamber (Figure 6b, black line). A relatively low desorption of HCHO gas was observed in the hydrophilic BAF (KGM-9%) (Figure 6b, red line). Since the hydrophilic BAF (KGM-9%) consists of KGM containing 11 hydroxyl groups per repeating unit, the low desorption of HCHO gas could be attributed to the abundant hydroxyl groups in the hydrophilic BAF (KGM-9%), which was further confirmed by the hydrophilic BAF (KGM-33%) case. No desorption of HCHO gas was observed in the hydrophilic BAF (KGM-33%), while additional adsorption of HCHO gas was observed. These results indicated that the hydrophilic BAF (KGM-33%), which possessed more abundant hydroxyl groups than the hydrophilic BAF (KGM-9%), could stably and tightly capture more HCHO gas without any desorption of HCHO gas.

HCHO gas could be polymerized to para-HCHO due to its instability when HCHO gas was adsorbed onto the hydroxyl groups of hydrophilic BAF [32,33]. The para-HCHO could not desorb from the surface of the hydrophilic BAF due to the size of the para-HCHO, even when pumped under vacuum at room temperature. These results suggest that the HCHO adsorbed on the hydrophilic BAF would not desorb during daily use. According to the guidelines of the World Health Organization (WHO) [34], the occupational exposure limit of HCHO gas is 0.5 ppm per 8 h. Hydrophilic BAF (KGM-33%) maintains HCHO concentrations of 0.41–0.31 ppm at 60–150 min, which is below the WHO guidelines (Figure 6b, green line). However, in the absence of an air filter, the concentrations of HCHO gas were 0.7–0.92 ppm even under strong air circulation, which exceeded the WHO guidelines (Figure 6b, black line). Previous reports did not meet the needs of real situations because extremely high HCHO concentrations of several hundred to a thousand ppm are generally used for HCHO adsorption tests [32,33]. The abovementioned results demonstrated that hydrophilic BAF (KGM-33%) was useful for practical concentration ranges of HCHO exposure (<5 ppm) in daily life. To confirm the adsorption of HCHO gas onto the hydrophilic BAF (KGM-33%), FT-IR spectroscopy was performed. The decrease in the -OH stretching band at 3233 cm^−1^ suggested the adsorption of HCHO onto the hydroxyl group of the hydrophilic BAF (red line) [15,32,33]. Six vibrational bands at 1539 cm^−1^ (C=O stretching), 1441 cm^−1^ (-CH stretching), 1326 cm^−1^ (C-O stretching), 1086 cm^−1^ (C-O-C stretching), 1078 cm^−1^ (C-OH stretching), and 809 cm^−1^ (CH stretching), increased in intensity and were attributed to the formation of para-HCHO (Figure 6c, red line) [15,32,33]. FT-IR data demonstrated transformation of the HCHO gas into para-HCHO in the hydrophilic BAF.

## 4. Conclusions

Hydrophilic and hydrophobic BAFs were synthesized through the physical etching of a cylindrical MFS, followed by one-step coating of the MFS bead with PVA-KGM and PDMS, respectively. Hybrid BAFs were prepared by combining hydrophilic and hydrophobic BAFs. A tower air filtration system in which BAFs were actively rotated by a fan motor to remove PM or HCHO gas was designed. A tower air filtration system loaded with the hybrid BAF-200 purified 3.63 L of PM (2500 μg/m^3^ PM_2.5_) at a high flow rate of 3.4 m/s with high removal efficiency (99.4% for PM_2.5_) and a low pressure drop (19 Pa) over 6 min. Since hybrid BAF-200 possessed both a high-dipole moment due to the hydrophilic BAF-100 and a strong electric field due to the hydrophobic BAF-100, PM was effectively removed by both substituents. The pressure drop (19 Pa) was much lower than those of previously reported filters, even at a high air velocity of an order of magnitude higher than those previously reported. Against our expectations, the PM_2.5_ removal efficiency slightly increased as the air velocity increased, suggesting that the removal efficiency could be enhanced even at high air velocities because the chance of contact between the glass chamber and the BAFs increased, leading to the formation of a stronger electric field. The hybrid BAF-200 showed excellent recyclability of up to 50 cycles with high removal efficiencies (99.4–93.4% for PM_2.5_). Furthermore, hydrophilic BAF-200 could permanently remove 3.73 L of HCHO gas (4.87 ppm) and return the ambient HCHO to safe levels (0.41–0.31 ppm) within 60 min without any desorption of HCHO gas.

## Data Availability

Not applicable.

## Supplementary Materials

The following are available online at https://www.mdpi.com/article/10.3390/polym14030422/s1. Figure S1: Images of 4 mm- and 9 mm-sized bead MFS sponges. Figure S2: (Top) SEM Image of MFS and (bottom) its corresponding EDX data. Figure S3: Image of a tower air filtration system equipped with a differential pressure gauge. Figure S4: Images of air filtration systems loaded with (a) BAF-300 and (b) BAF-400. Figure S5: PM_2.5_ concentration variations of hydrophilic BAF-100 to 400 as a function of increasing time. PM_2.5_ concentration variations of hydrophilic, hydrophobic, and hybrid BAF-200 as a function of increasing time. PM_2.5_ concentration variations of the fixed BAFs (hybrid BAF-200) and movable BAFs (hybrid BAF-200) as a function of increasing time. Figure S6: Images of hybrid BAF-200 and hydrophobic BAF-200 after operation. Figure S7: Image of the hybrid BAF-200 fixed at the bottom section of the air filter chamber. Figure S8: (a) PM2.5 removal efficiencies of fixed air filters. (b) PM2.5 concentration variations of fixed air filters as a function of increasing time. Figure S9: (a) BET nitrogen adsorption-desorption isotherm of hydrophilic BAFs (MFS/PVA-KGM) and hydrophobic BAFs (MFS/PDMS). (b) BET surface areas of hydrophilic BAFs, hydrophobic BAFs, and hybrid BAFs. Figure S10: Weight variations of hybrid BAF-200 up to 50 cycles. Figure S11: Net HCHO removal (a) efficiencies and (b) capacities of hydrophilic BAF (KGM-33%) and BAF (KGM-9%).
