# Supplementary material for: Hybrid Bead Air Filters with Low Pressure Drops at a High Flow Rate for the Removal of Particulate Matter and HCHO
Source: Polymers (Basel). 2022 Jan 21;14(3):422. doi: 10.3390/polym14030422 (PMC8840364; doi:10.3390/polym14030422)
Supplement: Supplementary file 1 [file polymers-14-00422-s001.zip › polymers-1493873-supplementary.pdf]

polymers

MDPI

Supporting Information

Hybrid Bead Air Filters with Low Pressure Drops at a High Flow Rate for the Removal of Particulate Matter and HCHO

Hee Ju Kim [1], Ye Jin Kim [1], Yu Jin Seo [1], Ji Hee Choi [1], Hye Young Koo [2] and Won San Choi [1,*]

1 Department of Chemical and Biological Engineering, Hanbat National University, 125 Dongseodaero, Yuseong-gu, Daejeon 305-719, Korea; kimhj0924@naver.com (H.J.K.); agoqkfkrl@naver.com (Y.J.K.); 6285999@naver.com (Y.J.S.); wlgml1350@naver.com (J.H.C.)

2 Functional Composite Materials Research Center, Jeonbuk Institute of Advanced Composite Materials, Korea Institute of Science and Technology (KIST), 92 Chudong-ro, Bongdong-eup, Wanju-gun, Jeollabuk-do, Seoul 136-791, Korea; koohy@kist.re.kr

* Correspondence: choiws@hanbat.ac.kr; Tel.:+82-42-821-1540

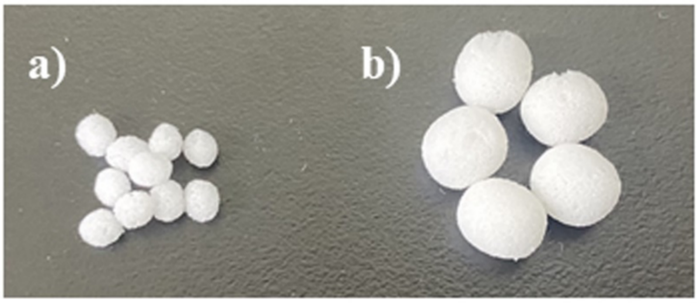

Figure S1. Images of (a) 4 mm and (b) 9 mm-sized bead MFS sponges.

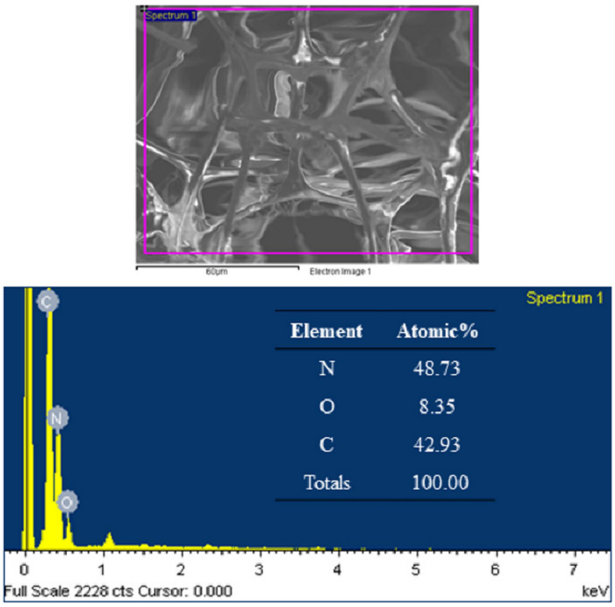

Figure S2. (Top) SEM Image of MFS and (bottom) its corresponding EDX data.

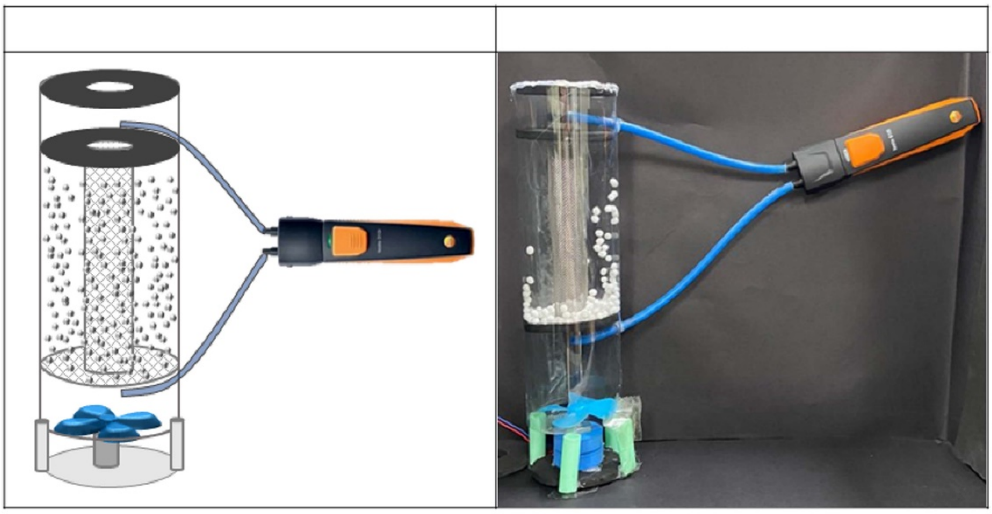

Figure S3. Image of a tower air filtration system equipped with a differential pressure gauge.

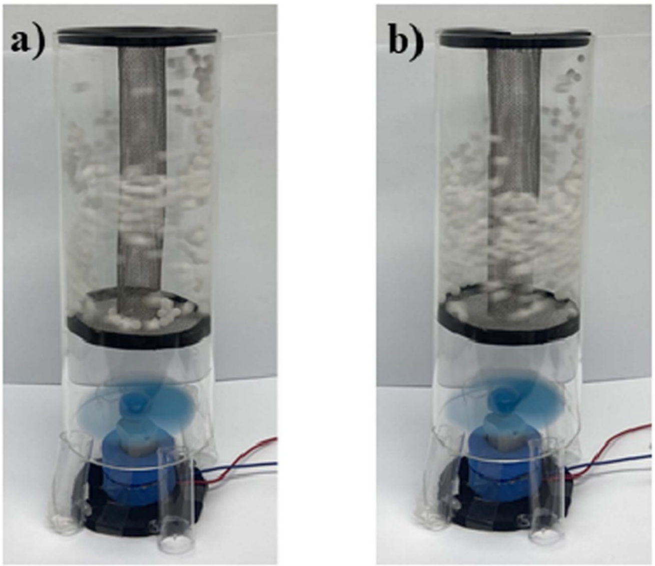

Figure S4. Images of air filtration systems loaded with (a) BAF-300 and (b) BAF-400.

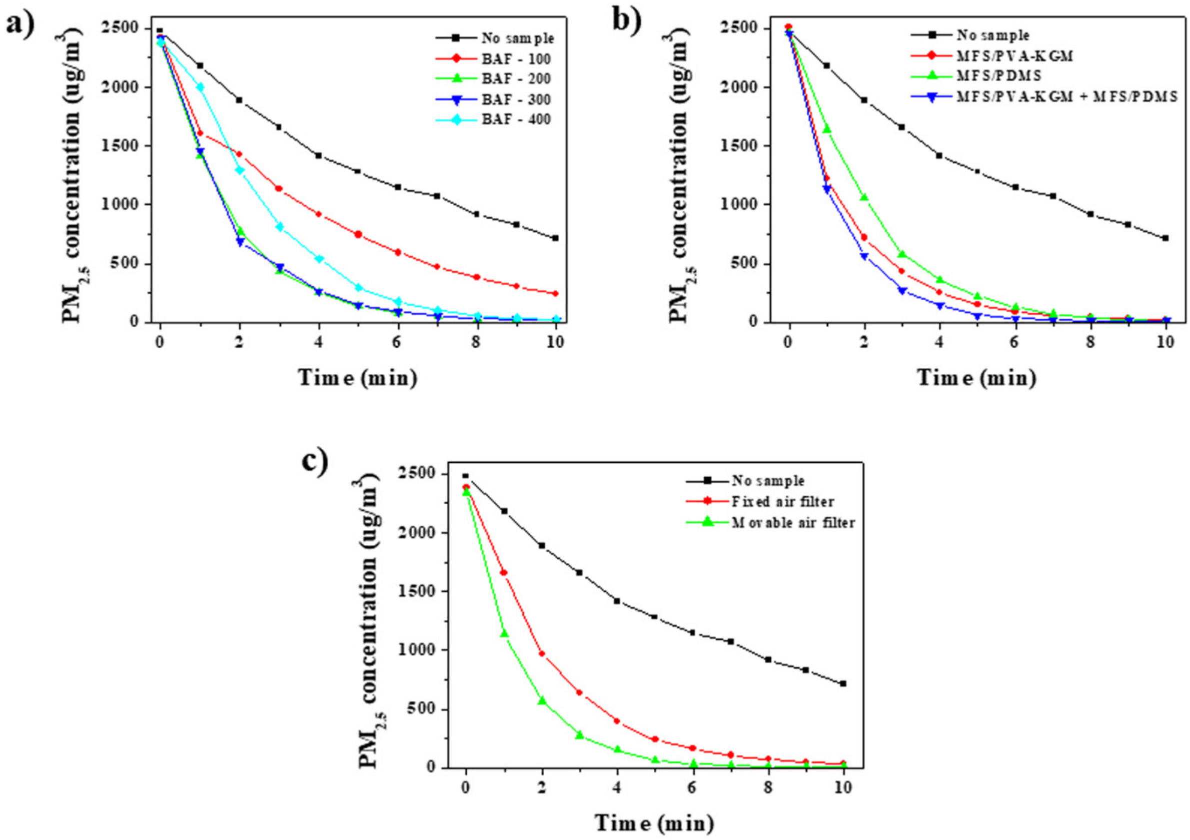

Figure S5. (a) $PM_{2.5}$ concentration variations of hydrophilic BAF-100 to 400 as a function of increasing time. (b) $PM_{2.5}$ concentration variations of hydrophilic, hydrophobic, and hybrid BAF-200 as a function of increasing time. (c) $PM_{2.5}$ concentration variations of the fixed BAFs (hybrid BAF-200) and movable BAFs (hybrid BAF-200) as a function of increasing time.

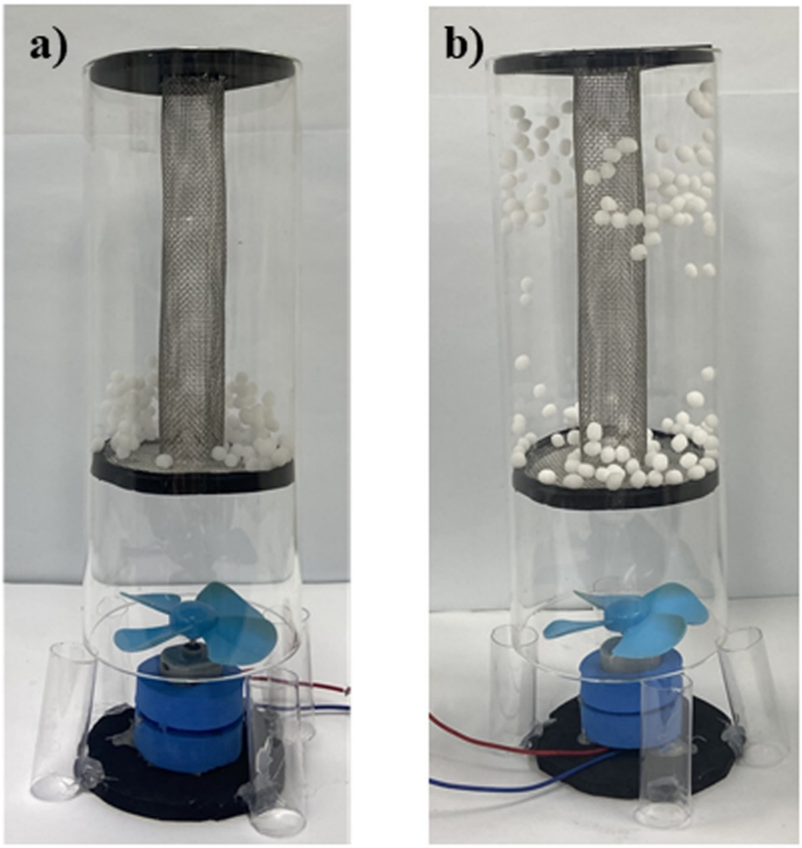

Figure S6. Images of (a) hybrid BAF-200 and (b) hydrophobic BAF-200 after operation (rotational motion of BAFs within the glass chamber).

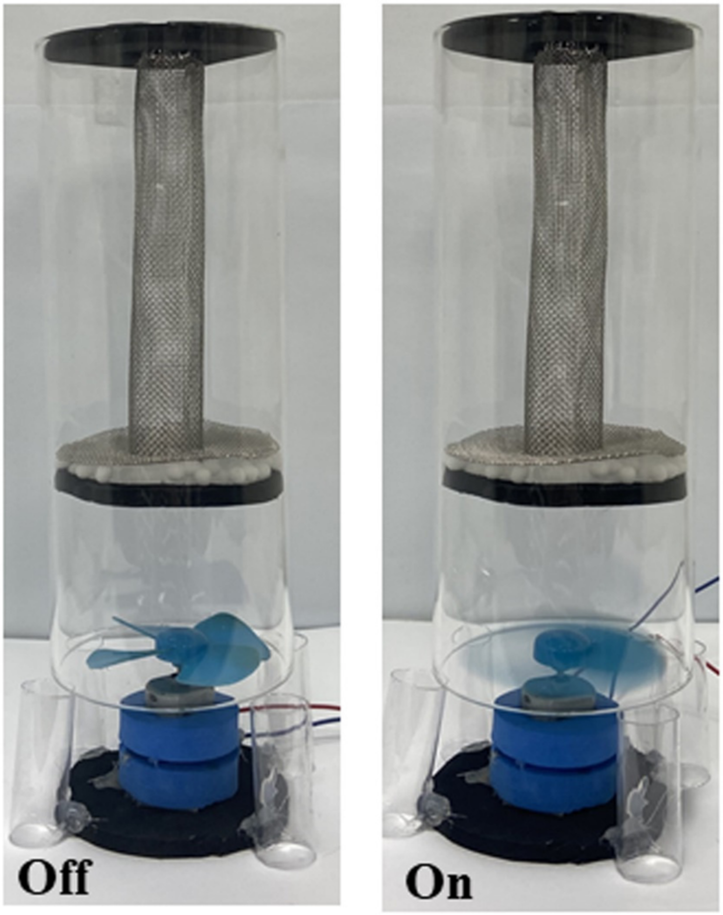

Figure S7. Image of the hybrid BAF-200 fixed at the bottom section of the air filter chamber.

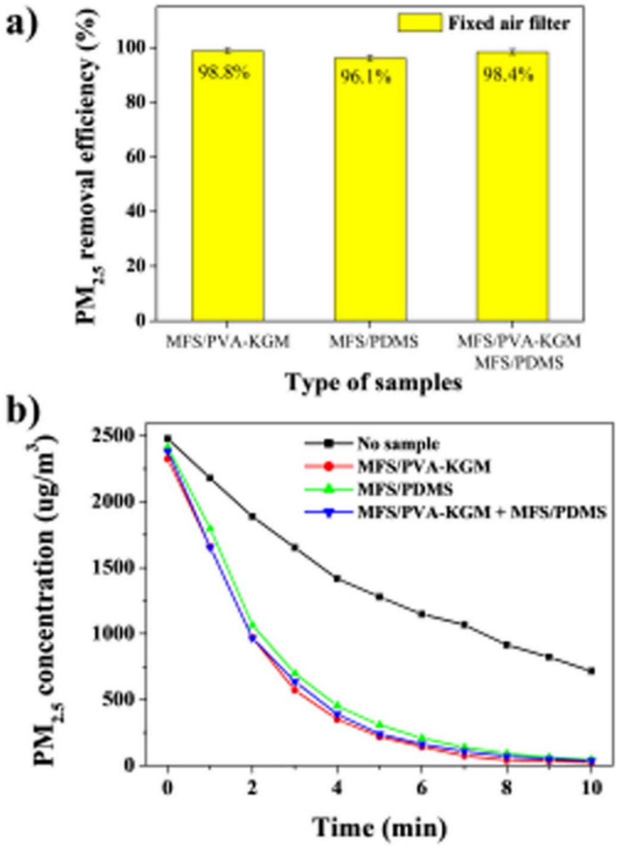

Figure S8. (a) $PM_{2.5}$ removal efficiencies of fixed air filters. (b) $PM_{2.5}$ concentration variations of fixed air filters as a function of increasing time.

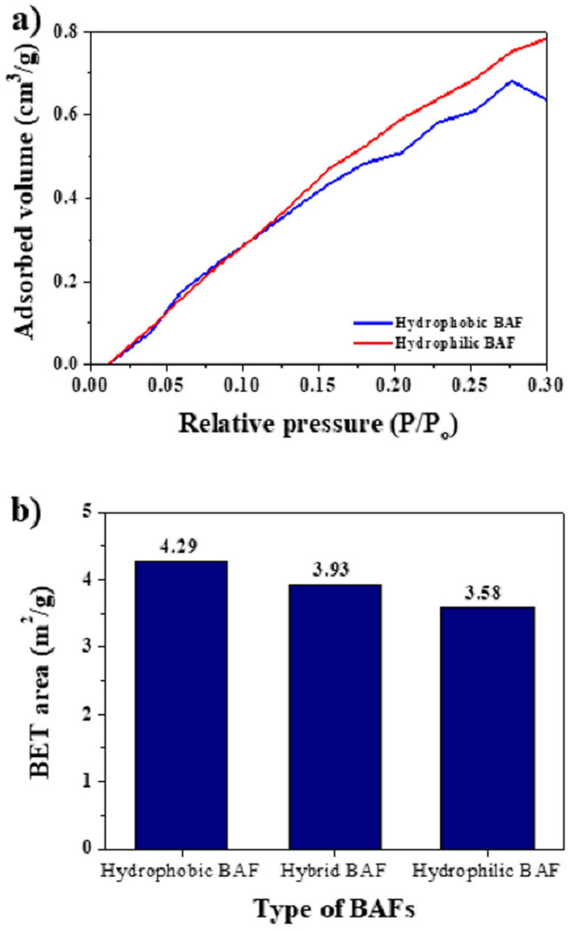

Figure S9. (a) BET nitrogen adsorption-desorption isotherm of hydrophilic BAFs (MFS/PVA-KGM) and hydrophobic BAFs (MFS/PDMS). (b) BET surface areas of hydrophilic BAFs, hydrophobic BAFs, and hybrid BAFs.

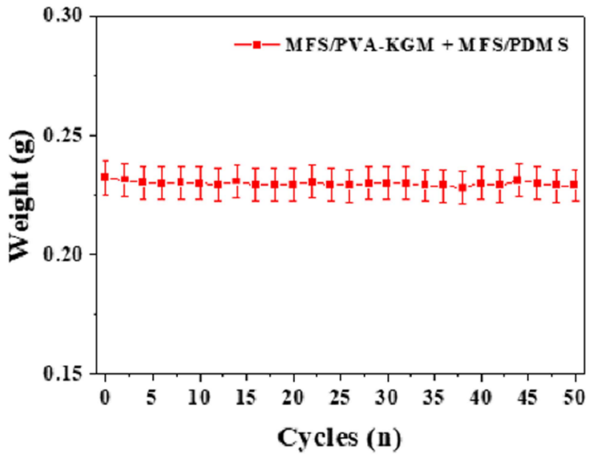

Figure S10. Weight variations of hybrid BAF-200 up to 50 cycles.

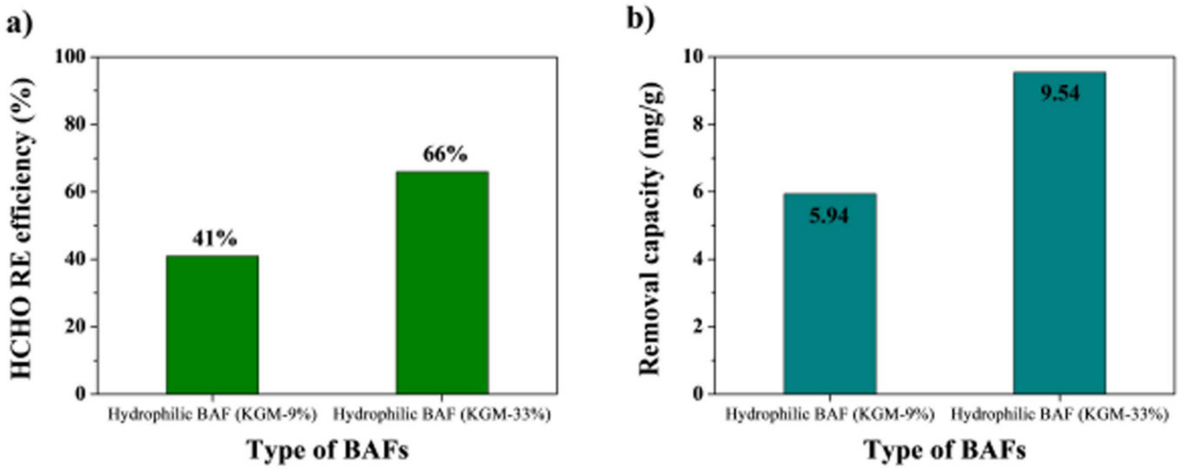

Figure S11. Net HCHO removal (a) efficiencies and (b) capacities of hydrophilic BAF (KGM-33%) and BAF (KGM-9%).

By comparing the HCHO concentrations with (C_1) and without filters (C_0) at 130 min, the net removal efficiency (RE) can be calculated according to the following equation:

$$\text{Net RE (\%)} = (C_0 - C_1)/C_0 \times 100\% \quad \text{(S1)}$$

where C_1 (mg L^{-1}) and C_0 (mg L^{-1}) refer to the HCHO concentrations with (C_1) and without filters (C_0) at 130 min, respectively.
